# The Effect of Predeformation on Creep Strength of 9% Cr Steel

**DOI:** 10.3390/ma13235330

**Published:** 2020-11-25

**Authors:** Petr Král, Jiří Dvořák, Wolfgang Blum, Václav Sklenička, Zenji Horita, Yoichi Takizawa, Yongpeng Tang, Lenka Kunčická, Radim Kocich, Marie Kvapilová, Marie Svobodová

**Affiliations:** 1 Institute of Physics of Materials, Academy of Sciences of the Czech Republic, Zizkova 22, 616 62 Brno, Czech Republic; dvorak@ipm.cz (J.D.); vsklen@ipm.cz (V.S.); kuncicka@ipm.cz (L.K.); kvapilova@ipm.cz (M.K.); 2 Department of Materials Science, Institute I, University of Erlangen-Nuremberg, D-91058 Erlangen, Germany; wolfgang.blum@fau.de; 3 Department of Materials Science, Kyushu Institute of Technology, Kitakyushu 804-8550, Japan; horita.zenji.688@m.kyushu-u.ac.jp; 4 Magnesium Research Center, Kumamoto University, Kumamoto 860-8555, Japan; 5 Synchrotron Light Application Center, Saga University, Saga 840-8502, Japan; 6 Technology Department, Nagano Forging Co., Ltd., Nagano 381-0003, Japan; ytakizawa@nsc-com.co.jp; 7 World Premier International Research Initiative, International Institute for Carbon-Neutral Energy Research (WPI-I2CNER), Kyushu University, Fukuoka 819-0395, Japan; tang.yongpeng.373@m.kyushu-u.ac.jp; 8 Faculty of Materials Science and Technology, Technical University of Ostrava, 17. Listopadu 15, 708 00 Ostrava, Czech Republic; radim.kocich@vsb.cz; 9 UJP PRAHA a.s., 156 10 Praha-Zbraslav, Czech Republic; svobodova@ujp.cz

**Keywords:** creep-resistant 9% Cr steels, severe plastic deformation, microstructure

## Abstract

Martensitic creep-resistant P92 steel was deformed by different methods of severe plastic deformation such as rotation swaging, high-pressure sliding, and high-pressure torsion at room temperature. These methods imposed significantly different equivalent plastic strains of about 1–30. It was found that rotation swaging led to formation of heterogeneous microstructures with elongated grains where low-angle grain boundaries predominated. Other methods led to formation of ultrafine-grained (UFG) microstructures with high frequency of high-angle grain boundaries. Constant load tensile creep tests at 873 K and initial stresses in the range of 50 to 300 MPa revealed that the specimens processed by rotation swaging exhibited one order of magnitude lower minimum creep rate compared to standard P92 steel. By contrast, UFG P92 steel is significantly softer than standard P92 steel, but differences in their strengths decrease with increasing stress. Microstructural results suggest that creep behavior of P92 steel processed by severe plastic deformation is influenced by the frequency of high-angle grain boundaries and grain coarsening during creep.

## 1. Introduction

Slight predeformation (up to 0.2) of materials can cause both improvement and deterioration of creep properties [1]. Investigation of creep behavior of predeformed heat resistant austenitic steels revealed that the effect of predeformation on creep behavior depends on deformation mode. It was demonstrated that predeformation in tension leads to improvement of creep resistance [2]. However, the opposite effect was found after application of compression predeformation [1]. Deterioration of creep properties was also observed in 2.25Cr–1Mo ferritic steel processed by tension predeformation at 873 K. Kikuchi and Ilschner [3] investigated the effect of 1% predeformation at different temperatures on creep and revealed that the higher the predeformation temperature, the smaller the difference in creep resistance with respect to the undeformed state.

Similar results were found in P92 steel processed by hot bending at 1193–1233 K and subsequent creep at 873 K. It was shown [4] that hot bending caused insignificant differences in creep strength falling within a scatter band of the unbent pipe. However, insignificant differences in creep strength of 9% of Cr steels were observed after cold bending [5].

F. Abe [6] investigated creep behavior of P91 steel at 873 K and 78 MPa after cold rolling with plastic strains of 0.2 and 0.4. It was found that time to fracture decreases with increasing cold rolling deformation.

Current methods of severe plastic deformation (SPD) allow significantly larger strain to be imposed in comparison with standard forming methods such as rolling or bending [7,8,9]. In the case where extremely large deformation is imposed to the material, transformation of a coarse-grained structure to an ultrafine-grained one (grain size 0.1–1 μm) occurs [7]. Nevertheless, transformation of the microstructure using SPD methods proceeds subsequently. At the beginning of the transformation, the microstructure contains, in particular, cell walls containing tangles with very high dislocation density. The further deformation leads to subsequent increasing of misorientation between cells and formation of an ultrafine-grained microstructure. Thus, the microstructures formed by SPD contain various proportions of low-angle (LA) and high-angle (HA) grain boundaries (GB), depending on the level of imposed plastic strain. For this reason, SPD techniques provide a unique opportunity to study the influence of significantly different microstructures on creep behavior of metals. Recent results showed that differences in the creep strength of SPD-processed state and its undeformed counterpart depend on the value of plastic strain imposed into the material [10,11,12,13,14], temperature, and applied stress used during creep testing [15,16,17].

The aim of this work is to study the influence of predeformation on creep strength and the microstructure of P92 processed by severe plastic deformation (SPD), with equivalent strains ranging from 1 to 20.

## 2. Materials and Methods

The experimental material used in the present work was advanced tungsten-modified martensitic 9%Cr P92 steel. The chemical composition of P92 steel (wt.%) was as follows: 0.11C, 8.58Cr, 0.33Mo, 1.67W, 0.37Si, 0.48Mn, 0.23V, 0.06Nb, 0.013P, 0.037N, 0.005S, 0.0015B, and 0.017Al. Heat treatment of the as-received coarse-grained (CG) state consisted of normalization at 1323 K for 60 min, followed by tempering at 1013 K for 140 min [4].

Discs of 30 mm diameter and 1.1 mm thickness and sheets with dimensions of 10 × 100 × 1.1 mm^3^ were cut from the as-received and relatively coarse-grained P92 steel (CG). The discs were processed by 1 rotation high-pressure torsion (HPT) at room temperature under the pressure of 6 GPa and rotation speed of 0.1 mm per second. The sheets were processed by high-pressure sliding (HPS) at room temperature with a sliding distance of 5 mm (HPS5) and 15 mm (HPS15) under the pressure of 4 GPa. The value of von Mises equivalent strain during HPT [7] grows with the distance, *r*, from the center of the disc according to $\varepsilon_{\mathrm{SPD}}=2\pi rN/\sqrt{3}t$ where *N* is the number of turns and *t* is the thickness of disc. The value of von Mises equivalent strain during the HPS process [18] was estimated as $\varepsilon_{\mathrm{SPD}}=\frac{x}{\sqrt{3}t},$ where *x* is the sliding distance of the plunger with respect to the anvil and *t* is the thickness of the sample.

The equivalent strain imposed by rotation swaging (RS) [19,20] was estimated by *ε*_SPD_ = ln(*D*_0_/*D*_n_)^2^, where *D*_0_ is the initial diameter ~30 mm and *D*_n_ is the final diameter ~15 mm after application of RS at room temperature. The values of equivalent strain *ε*_SPD_ imposed by selected methods of SPD are shown in Table 1.

Constant load tensile creep tests were conducted at 873 K (~0.48 of melting temperature) in protective argon atmosphere using flat specimens with a gauge length of 10 mm and a cross section of 3 × 1 mm^2^. The tested HPT specimens were manufactured from the disc region with equivalent strain of about 25 ± 5 [12,21]. The gauge lengths of the HPS tensile specimens were taken from the central part of the sheet.

Microstructure investigations were performed in a scanning electron microscope (SEM, Tescan Lyra 3, (Brno, Czech Republic), equipped with NordlysNano EBSD (High Wycombe, UK) detector operating at accelerating voltage of 20 kV with specimen tilted at 70°) and a transmission electron microscope (TEM, JEOL 2100F, Tokyo, Japan). SEM was used to determine the misorientations *θ* between neighboring grains. *θ* = 15° was taken to distinguish HAGBs from LAGBs consistent with previous works [10,11,12]. The mean spacing of LAGBs along test lines is called *w*, and the mean spacing of HAGBs is called *d*. The fraction of HAGBs is estimated as *f*_HAGB_ = *w*/*d*.

## 3. Results

### 3.1. Microstructure before Creep

**Figure 1:**
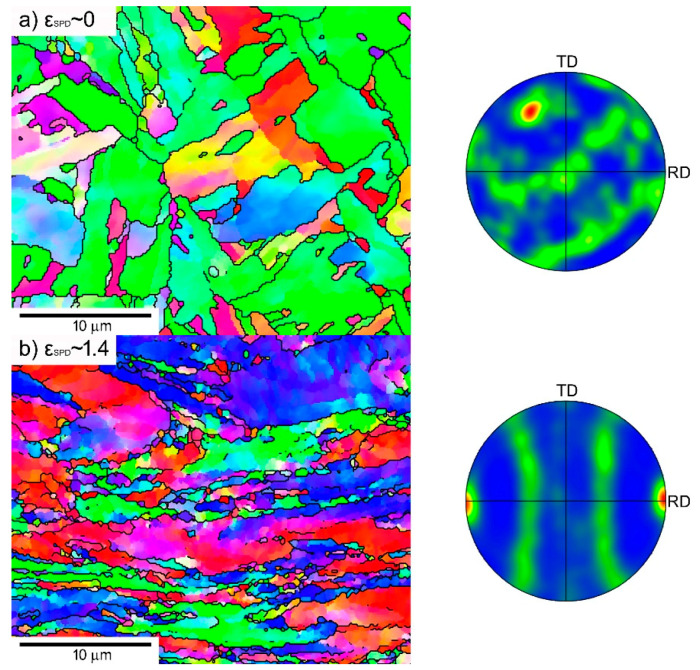

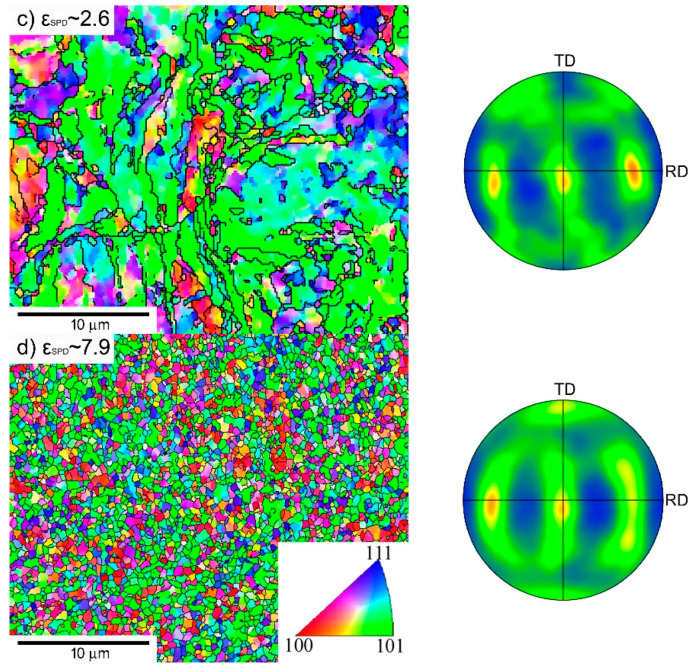
Grain microstructure (orientation contrast with high-angle grain boundaries (HAGB) only, normal direction (ND) and texture (poles of {110} planes) at room temperature before creep for (**a**) as-received coarse-grained (CG), (**b**) rotation swaging (RS), and (**c**) high-pressure sliding, HPS5 and (**d**) HPS15; high-pressure torsion (HPT) is similar to HPS15. Rolling directions (RD) are parallel with the pipe axis in CG state and shear direction in SPD-processed states.

**Figure 2:**
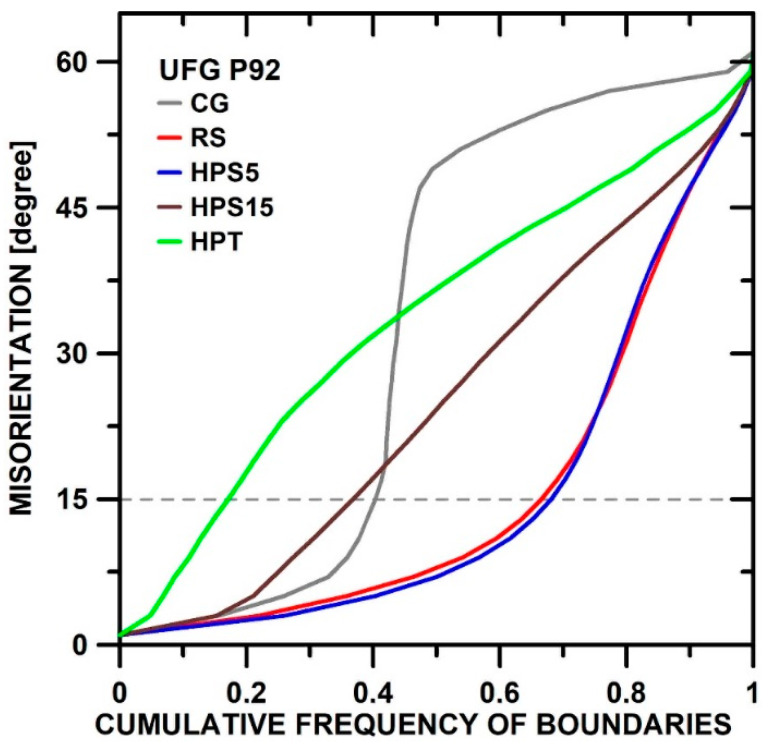
Misorientation: cumulative frequency *F* of boundaries with misorientations *θ* in CG (as-received) and predeformed P92. Intersection with the dashed line *θ* = 15° gives the low-angle grain boundaries (LAGB) fraction $f_{\mathrm{HAGB}}=F(15{}^{\circ})$.

**Figure 3:**
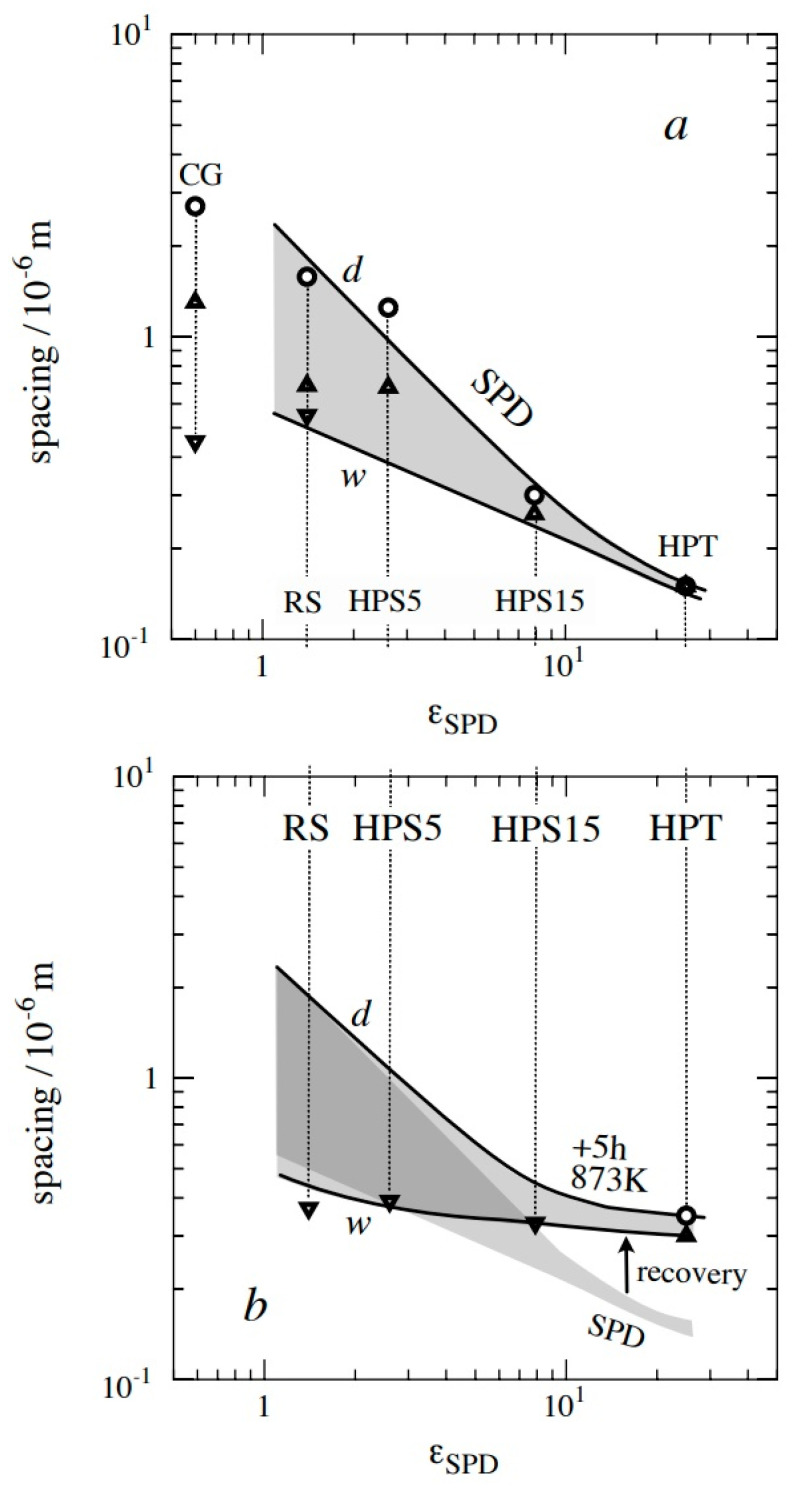
Boundary spacings *d* and *w* versus equivalent SPD strain (*ε*_SPD_) (**a**) after SPD (**b**) after annealing; CG data are displayed at *ε*_SPD_ = 0.6 for comparison reasons; the vertical extension of shaded area shrinks with increasing $f_{\mathrm{HAGB}}$.

In the following section we report microstructural details for the P92 variants investigated in this work regarding grains and their orientations (Figure 1), grain misorientations (Figure 2), and boundary spacings (Figure 3). They vary depending on the strain *ε*_SPD_ applied during SPD (Figure 3). As usual, the as-received state CG consists of prior austenite grains containing laths boundaries and other LA subgrain boundaries (not visible in Figure 1) in their interiors resulting from martensitic transformation. The microstructure of as-received state results from normalization at 1323 K for 60 min in air and subsequent tempering at 1013 K for 140 min.

RS has a heterogeneous grain structure with bands of fine nearly equiaxed submicron grains and large grains exceeding 30 μm that are significantly elongated parallel to the direction of swaging. The grain structure of HPS5 is also relatively coarse but more homogeneous. HPS15 has much more refined grains similar to HPT.

Figure 1 shows a slight tendency in CG to orientate {101} crystallographic planes nearly parallel to the pipe axis, which is identical to the stress axis during creep testing. The microstructure of the RS state contains a strong fiber texture {hkl}<101> parallel to the swaging axis (Figure 1b).

The HPS-processed specimens (Figure 1c,d) exhibited a fiber texture <110> perpendicular to the shear direction of HPS with {110}<111> and {110}<100> strongly pronounced variants. A similar texture was also observed in the HPT-processed specimens [21].

Figure 2 displays the cumulative frequency (F) of b oundaries with misorientations up to *θ*. For *θ* = 15°, *F* equals the LAGB fraction $f_{\mathrm{LAGB}}\equiv 1-f_{\mathrm{HAGB}}$. The steep inclination of the GC curve shows predominance of LAGBs with *θ* < 15° and HAGBs with high *θ* > 40° and a concentration near Σ 3 (60°/111). After SPD, such a gap in the distribution is missing. In RS and HPS5, *f*_HAGB_ is strongly raised by SPD-induced LAGB generation. With increase of *ε*_SPD_, strain-induced LABGs get converted into HAGBs, and *f*_HAGB_ increases to 0.85 for HPT.

Figure 3a visualizes the existing data for the mean boundary spacings in the investigated material variants after SPD. The *w*- and *d*-lines are not primarily precise fits of the limited results. The *w*-line with $w\propto 1/\varepsilon_{\mathrm{SPD}}^{0.5}$ is motivated by parabolic work hardening, $\sigma \propto \varepsilon_{\mathrm{SPD}}^{0.5}$, during SPD at room temperature toward the final stationary (saturation) stage and formation of quasi-stationary subgrain structures with $w_{\mathrm{qs}}\propto 1/\sigma$. The d-line with $d\propto 1/\varepsilon_{\mathrm{SPD}}$ decreases more strongly with $\varepsilon_{\mathrm{SPD}}$ than w because misorientation of θ-distribution shifts toward higher θ with $\varepsilon_{\mathrm{SPD}}$ by transformation of LAGBs into HAGBs. Vertical extension of the shaded area marks *f*_HAGB_; as the lines for *d* and *w* approach each other, *f*_LAGB_ shrinks and *f*_HAGB_ approaches 1.

Imposing *ε*_SPD_ = 1.4 by RS reduces the grain size *d* to 1.6 μm, and 35% of the boundaries are HAGBs (Figure 2) with relatively equal *θ*-distribution between 15–65°. Processing with HPS with a sliding distance of 5 mm to *ε*_SPD_ = 2.6 generates a grain size of about 1.25 μm and a similar portion of HAGBs. Imposing *ε*_SPD_ = 7.9 by HPS15 led to a significant decrease of mean grain size down to 0.35 μm and an increase of *f*_HAGB_ up to 0.7. The lowest grain size, *d*, and the highest, *f*_HAGB_, are found for HPT subjected to the highest SPD strain, *ε*_SPD_ = 25 ± 5.

However, grain parameters after SPD at room temperature are not the ones determining creep, as the grains coarsen during heating to the test temperature and subsequent soaking until the beginning of creep. While this annealing treatment before creep has little effect for micron-sized grains, it causes significant coarsening of submicron grains. This is seen in Figure 3b (see arrow “recovery”).

**Figure 4:**
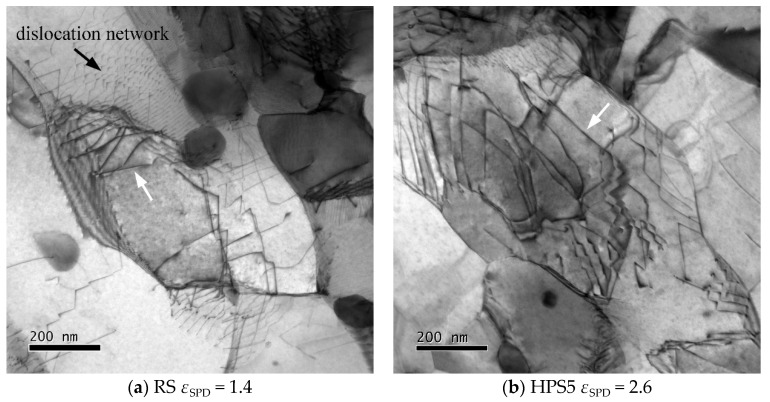

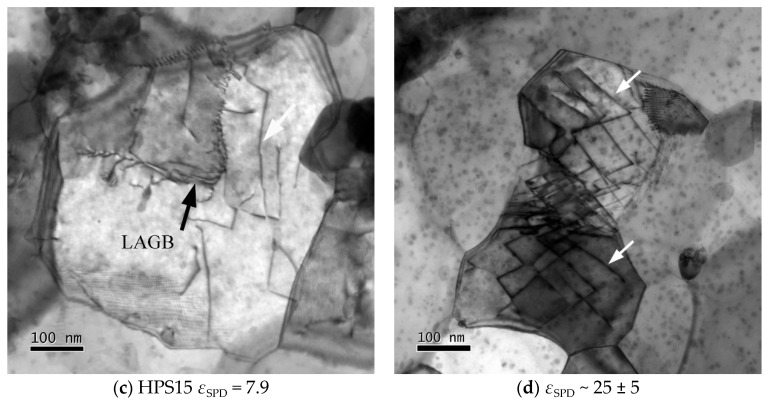
Microstructure (TEM) after annealing at 873 K for 5 h in (**a**) RS, (**b**) HPS5, (**c**) HPS15, and (**d**) HPT; note the higher magnification in (**c**,**d**).

**Figure 5:**
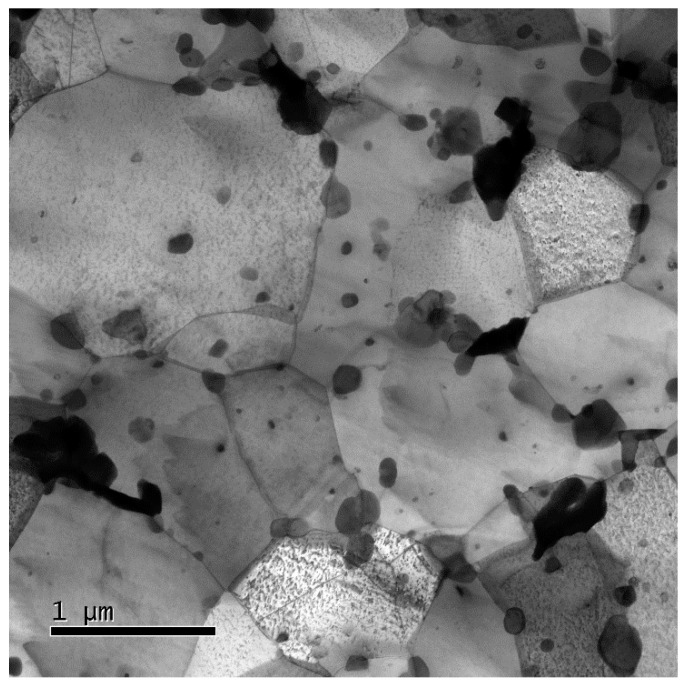
Microstructure of P92 steel processed by HPT and annealed at 923 K for 500 h.

Figure 4 shows the effect of annealing for 5 h at 873 K; this treatment simulates the annealing effect of heating to the test temperature. Despite different degrees of *ε*_SPD_ in RS, HPS5, HPS15, and HPT, the subgrain structures look qualitatively similar. Recovery of dislocation line length and boundary area involves motion of both free dislocations and boundaries to sites of recovery by recombination and annihilation. While this occurs, many relatively straight free dislocations (see arrows) are seen to extend between neighboring boundaries. The subgrain sizes *w* (mean intercepts) estimated from micrographs like those of Figure 5 are plotted in Figure 3b. The *w*- and *d*-coarsening is small for the relatively coarse grains of RS and HPS5, but it is prominent for the ultrafine grains of HPT where *d* goes up to 0.35 μm with a fraction of HAGB area (*f*_HAGB_) ~90%.

Even more (sub)grain coarsening occurs in annealing for 500 h at 923 K (Figure 5). Here the grains coarsen to *d* ~0.85 μm with *f*_HAGB_ ~87%. The high value of *f*_HAGB_ is understandable as no LABGs are formed during static annealing where no net plastic deformation occurs.

### 3.2. Creep Behavior

**Figure 6:**
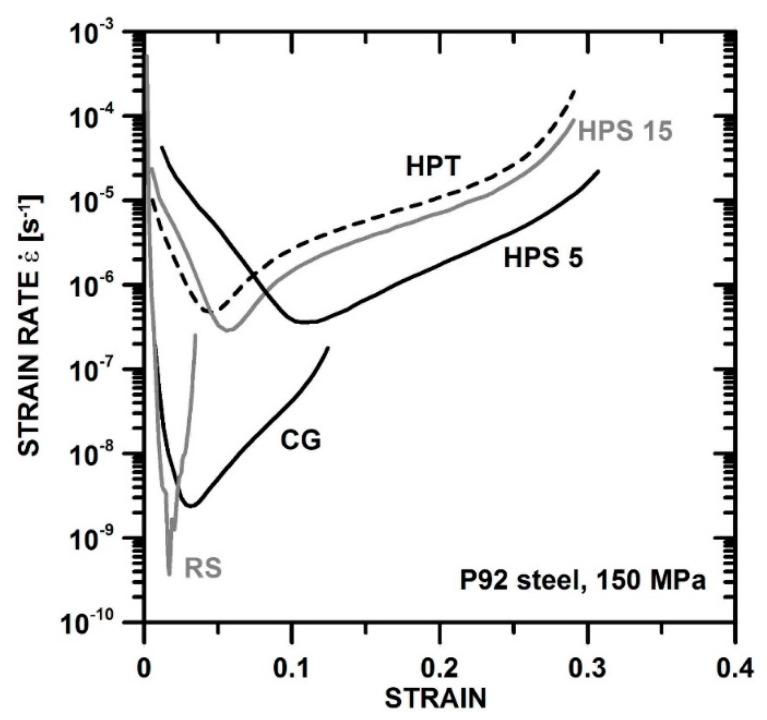
Evolution of strain rate with strain in tensile creep at constant load with *σ*_0_ = 150 MPa for different SPD strains *ε*_SPD_.

Figure 6 shows evolution of strain rate $\dot{\varepsilon }$ with strain in creep at *σ*_0_ = 150 MPa for all P92 variants. All curves display a pronounced relative minimum of creep rate ${\dot{\varepsilon }}_{\min}$ . HPT, HPS5, and HPS15 have similar ${\dot{\varepsilon }}_{\min}$. CG and RS creep much more slowly. Ductilities of different SPD specimens differ greatly. RS has the lowest ductility. The results will be discussed below in relation to microstructure.

**Figure 7:**
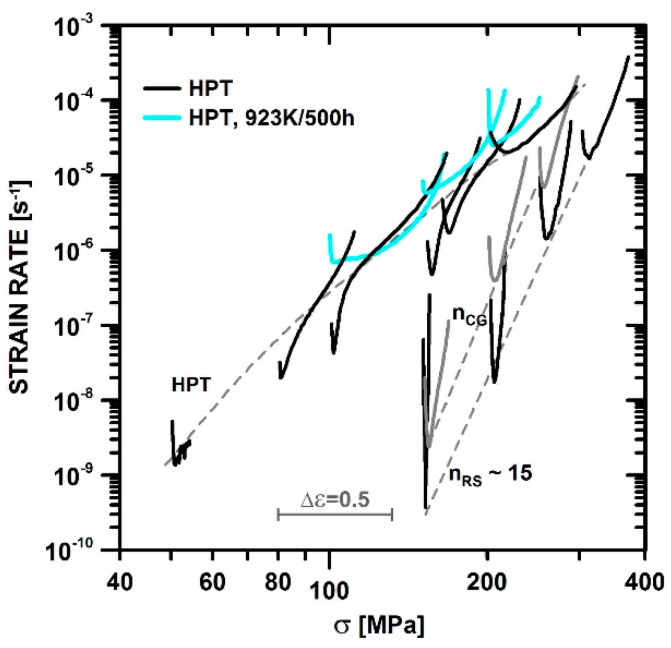
Creep rate–stress curves for HPT, CG, and RS.

Figure 7 shows the creep rates of CG, RS, and HPS versus stress. Tests at constant load have the advantage to show both the evolution with strain and the influence of stress in a single plot. This is because stress varies at constant load with strain, as

*σ* = *σ*_0_ exp(*ε*) (1)

provided that deformation is uniform. Empirically it is known that uniformity of deformation along the gauge length is provided in relatively ductile materials until the strain has reached about half the fracture strain [22]. According to Equation (1), the logarithmic stress axis corresponds to a linear strain axis.

Thus, the individual curves in Figure 7 can be viewed as $\dot{\varepsilon }$–ε curves like those of Figure 6 but shifted along the abscissa. Deformation begins at $\sigma =\sigma_{0}$; the scale bar marks an interval Δε = 0.5 of uniform strain. Not only do the minima of the curves displayed in Figure 7 show dependence on stress, the curves also show the full evolution of rate from the beginning, via strengthening and softening up to the second half of the curves where external and internal necking cause an over-exponential increase of rate until final fracture.

Figure 7 shows two main features. First, the global stress dependences of rates differ strongly for the variants HPS and HPT with the highest *ε*_SPD_ and the variants RS and CG with the lowest *ε*_SPD_. Roughly speaking, the stress exponent $n\;=dlog\dot{\varepsilon }/dlog\sigma$ of the rates is about 6 to 7 for HPS and HPT and twice that high for RS and CG. This large *n*-difference leads to the result that the minimum creep rates of all variants are similar at a high stress of about 400 MPa, while CG and RS creep much more slowly at the lower stress of 150 MPa. Figure 7 also displays softening during creep that is evident from the pronounced minimum due to the fact that the rate increases distinctly more strongly after the minimum than would be expected from stress sensitivity alone. In the literature, this steep increase is termed tertiary creep and is often linked to “damage” and fracture. However, if deformation is fairly uniform after the minimum, as in our ultrafine-grained (UFG) materials with fracture strains > 0.5, the rate increase must be attributed to softening by internal microstructural changes, e.g., coarsening processes (see Discussion) rather than fracture. The following subsection describes the observed changes.

### 3.3. Changes of Microstructure during Creep Testing

The microstructure of fractured tensile specimens was investigated at different locations along their length. In the grip parts, the local stress, *σ*_loc_, is negligible. In the gauge length, the stresses and strains vary with location. The local strain was roughly estimated as *ε*_loc_ = ln(*S*_loc_/*S*_0_) from the local cross section, *S*_loc_, and the initial cross section, *S*_0_. The local stress follows from Equation (1) as σ_loc_ = *σ*_0_ exp(*ε*_loc_).

**Figure 8:**
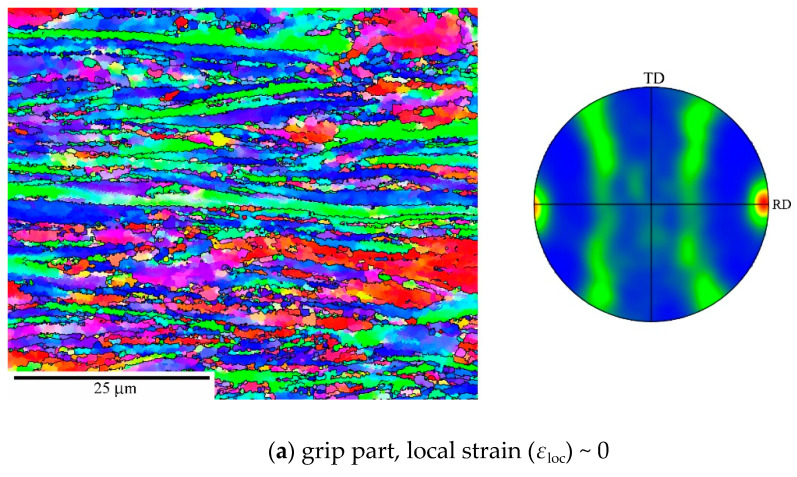

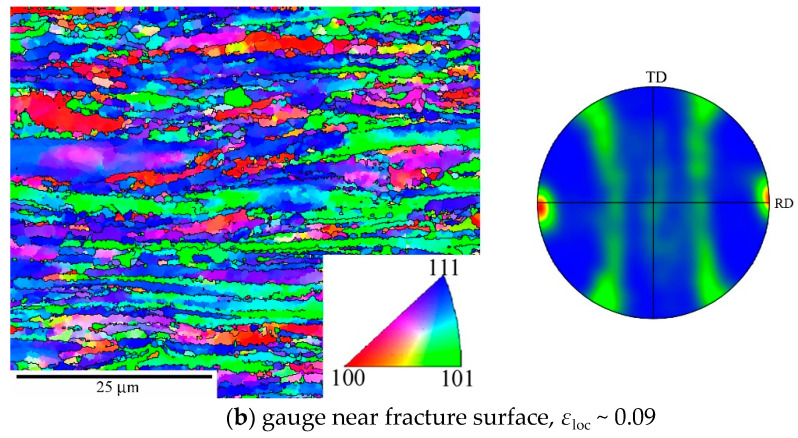
Microstructure and texture of RS after creep at *σ*_0_ = 200 MPa in (**a**) grip part and (**b**) gauge length near the fracture. RD directions are parallel with the stress axis.

**Figure 9:**
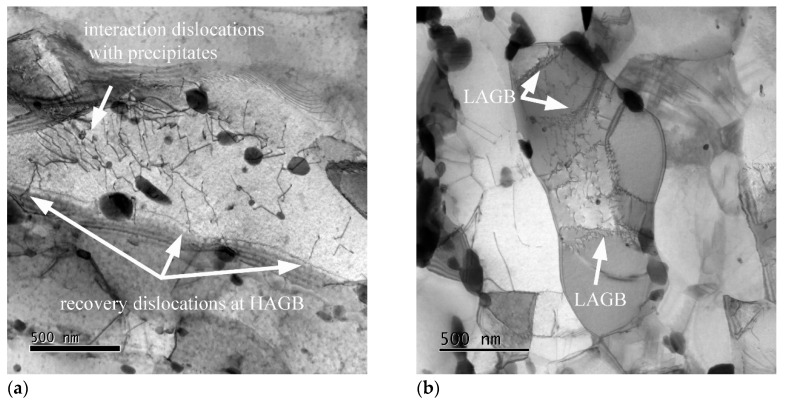
Dislocation microstructure in the gauge lengths of specimens tested: (**a**) 300 MPa and (**b**) 150 Mpa.

Figure 8 and Figure 9 show examples of the microstructure in SEM and TEM for the moderately predeformed variant RS after creep to fracture at different *σ*_0_ and different *σ*_loc_.

Grain structure and texture, subgrain, and dislocation structure look relatively similar to before creep (Figure 1b and Figure 4a). It is noted that the dislocation structures have all undergone recovery, either before creep or after fracture; therefore, Figure 9 probably is not representative for the dislocation structure in situ under creep stress.

**Figure 10:**
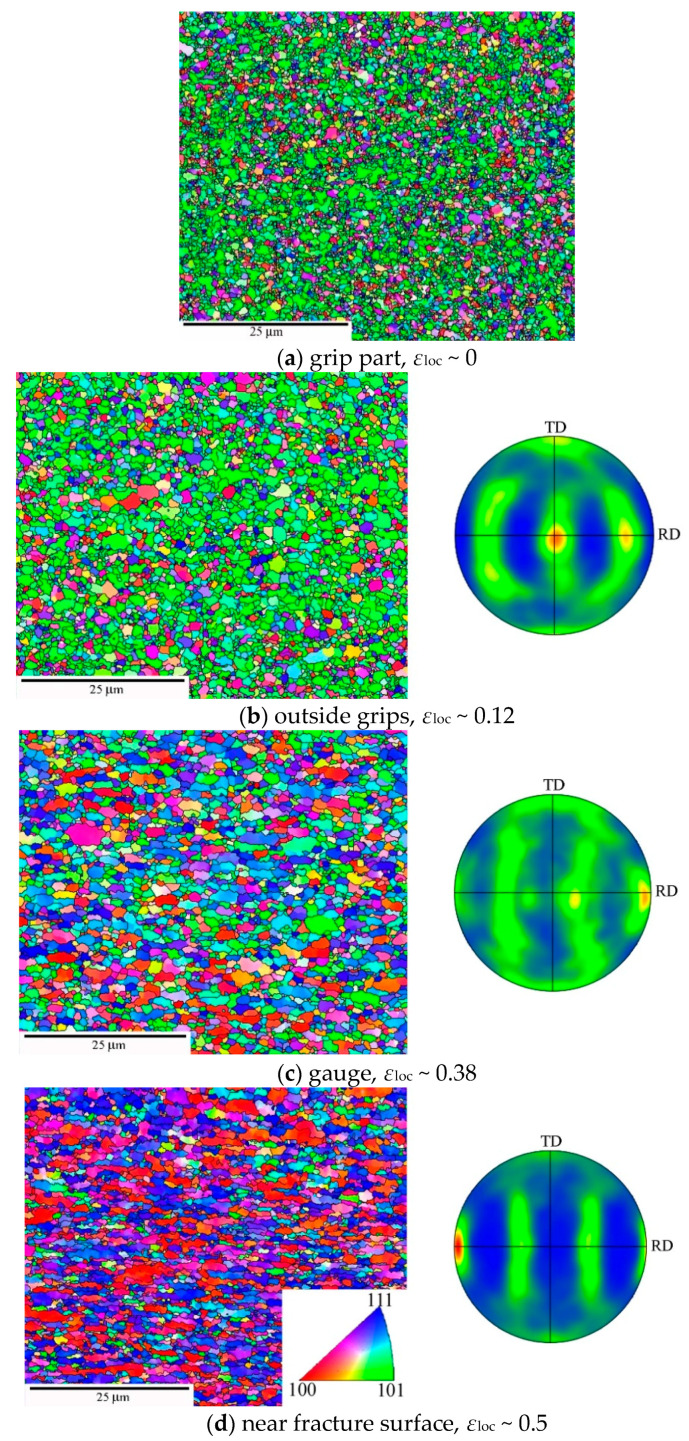
Grain structure and texture of HPS15 after creep at 160 MPa for ~7 h in grip and gauge with different *ε*_loc_. RD directions are parallel with the stress axis.

**Figure 11:**
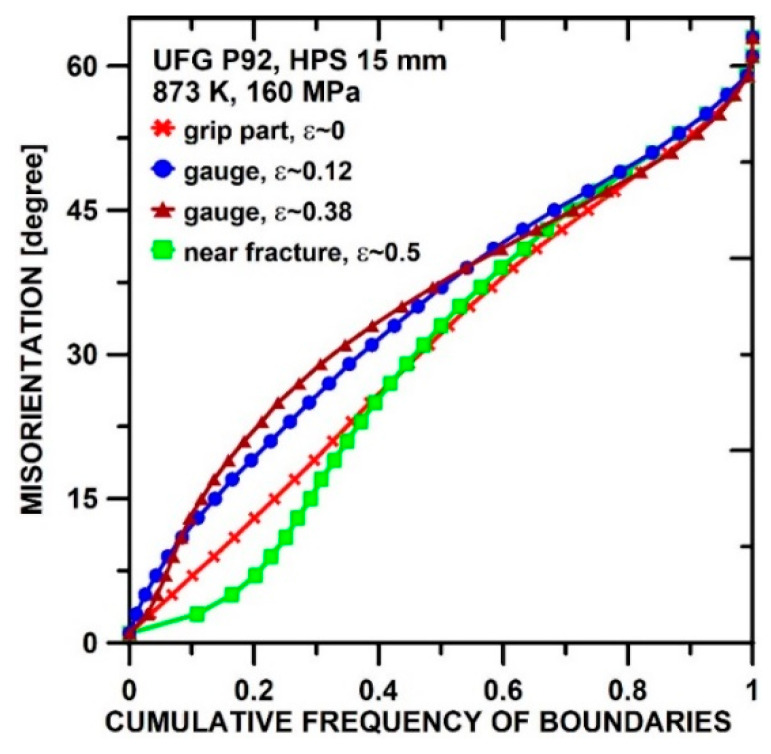
Evolution of misorientation distribution during creep of HPS15.

Next, we consider the most severely predeformed materials HPS15 and HPT. Figure 10 shows grain structure and texture for HPS15 in dependence of *ε*_loc_. Compared to the state before creep (Figure 4c), the ultrafine grains have coarsened statically in the grip part and even more so in gauge length. The texture did not change in the grip part and after slight creep strain in the gauge length (Figure 10a,b). The misorientation distribution evolves during creep (Figure 11). There is a significant increase of HAGB fraction *f*_HAGB_ during creep as *ε*_loc_ increases to 0.12 and 0.38. It appears that LAGBs migrate faster during grain coarsening by recovery through recombination with other boundaries than HAGBs do and so disappear earlier. Near the fracture surface, the HAGB fraction *f*_HAGB_ decreased again. This suggests massive deformation connected with formation of small subgrains in large grains at the high local stresses acting there.

**Figure 12:**
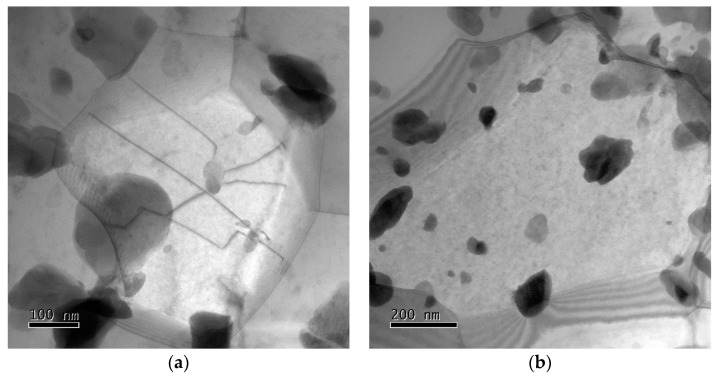

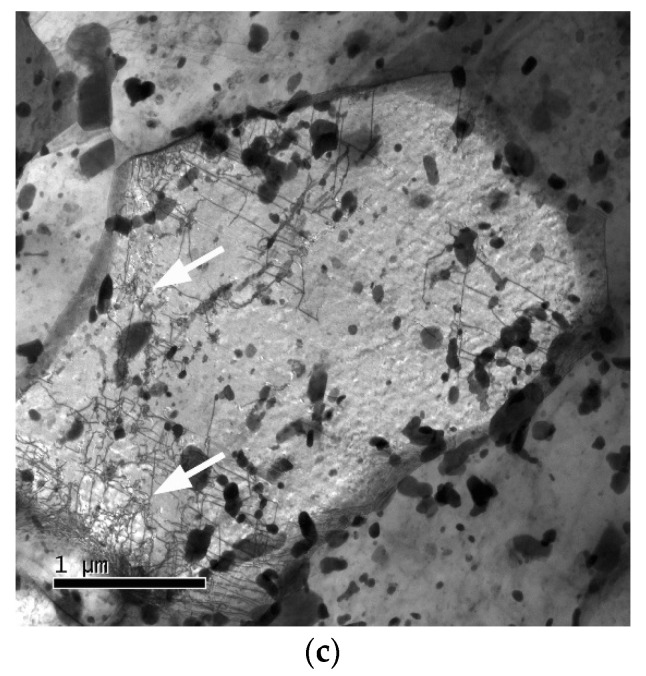
Dislocation microstructure in the HPS15-processed specimen tested at 873 K and 100 MPa in gauge length with (**a**) *ε*_loc_ ~0.03–0.05 (fine grain), (**b**) *ε*_loc_ ~0.03–0.05 (large grain), and (**c**) *ε*_loc_ ~ 0.2.

The TEM view of Figure 12 shows structural details in grains of different size. The ultrafine grains in Figure 12a,b are subgrain-free. The largest grain indicates considerable dislocation activity with formation of dislocation tangles as precursors of subgrains (see arrows). Precipitate particles are seen to be distributed over the grain interiors and at the boundaries.

**Figure 13:**
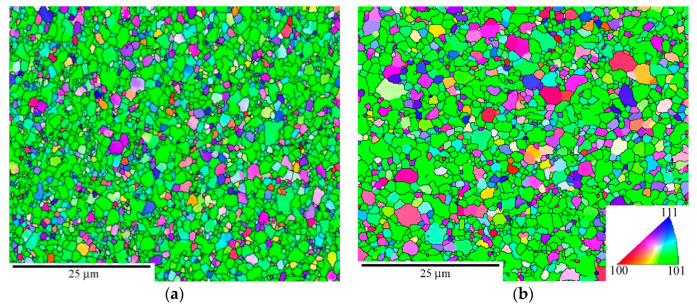
Grain structure of HPT with HAGB lines for *θ* > 15° after creep at *σ*_0_ = 50 MPa for 10,538 h (**a**) in the grip part and (**b**) in the gauge length at *ε* = 0.08. The stress axis is horizontal.

**Figure 14:**
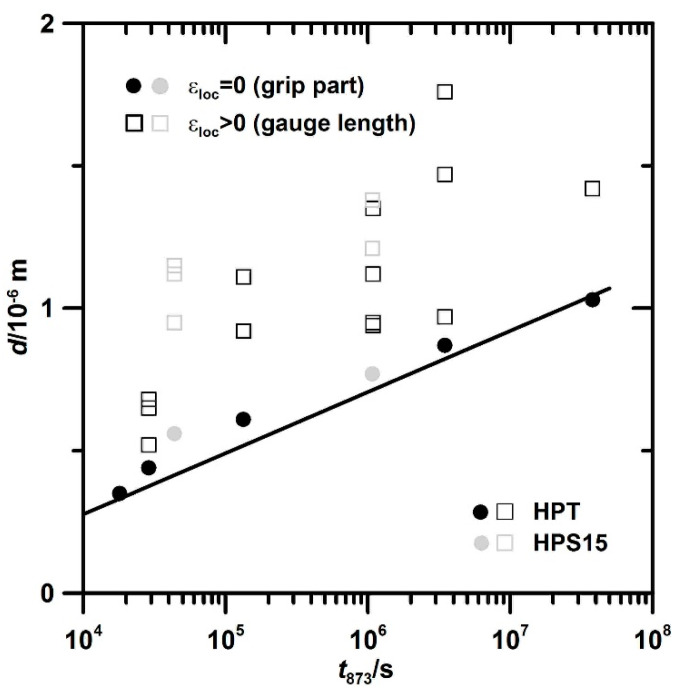
Increase of *d* with *t* during static growth in HPT specimens during annealing for 5 h at 873 K and subsequent creep at different stresses and times to fracture.

As the initial grain structures of HPT and HPS15 are similar at the beginning of creep (Figure 3b), similar evolution is to be expected. The slow creep test confirms grain coarsening (Figure 13). The small difference between grip (Figure 13a) and gauge (Figure 13b) confirms that coarsening is mainly static in nature under these conditions. The texture did not change during creep. Figure 14 presents grain coarsening in the grips of HPT specimens with time at test temperature. Limited grain growth during annealing before creep continues during creep. This follows from the continuous growth of *d* observed in the unstressed grips of different specimens. Coarsening during creep in the grips can empirically be described with sufficient accuracy by a straight fit line in the semi-log plot of Figure 14 given by:

*d* = *d*_ref_ + *k* log(*t*_873_/*t*_ref_) (2)

with *t*_ref_ = 1.3 × 10^4^ s, *d*_ref_ (*t*_ref_) = 3.01 × 10^−7^ m and k = 2.15 × 10^−7^ m.

## 4. Discussion

Deformation and fracture strengths of metals depend on obstacles to motion of free dislocation making the slipped areas grow. Obstacles include solutes, hard particles, and crystallite boundaries. The latter are present already in the as-received state due to thermomechanical treatment by martensitic transformation with a high degree of internal deformation and subsequent annealing. Additional thermomechanical treatment by SPD at room temperature, [7,8] plus static recovery during heating to elevated test temperature, has the potential to enhance the densities of individual dislocations and boundaries of LA- and HA-types. This would lead to strengthening in absence of recovery. However, in creep at elevated temperature, recovery plays a dominant role [23,24]. It makes a decisive difference.

After relatively small “primary” creep strain, deformation becomes quasi-stationary: free dislocations are generated but are also dynamically recovered. The balance of both processes is the main reason for the minimum of creep rate. Recovery controls how many dislocations can be pushed into the material. Therefore, the question for creep strength usually is the question for dynamic recovery of free dislocations and boundaries. Boundaries evolve relatively slowly with strain by migration-controlled recombination at triple points. Free dislocations evolve much faster. The creep around and after the minimum is quasi-stationary (qs) at relatively slowly evolving boundary structure. The boundaries act not only as obstacles for incoming free dislocations but also as sinks of free dislocations. Boundaries of LA-type tend to be weaker obstacles but less efficient recovery sites than boundaries of HA-type. At a given total boundary area ≈2/*w*, HAGBs dominate the quasi-stationary (qs) deformation resistance when their areal ratio *f*_HAGB_ ≈ *w*/*d* approaches 1.

This can explain why SPD-processed P92 was found to have lower creep resistance than as-received P92 [20] at low stresses. The other types of obstacles, solutes, and precipitates are expected to be more or less independent of SPD. Therefore, because of this and also shortage of data, we concentrate here on the influence of boundaries.

**Figure 15:**
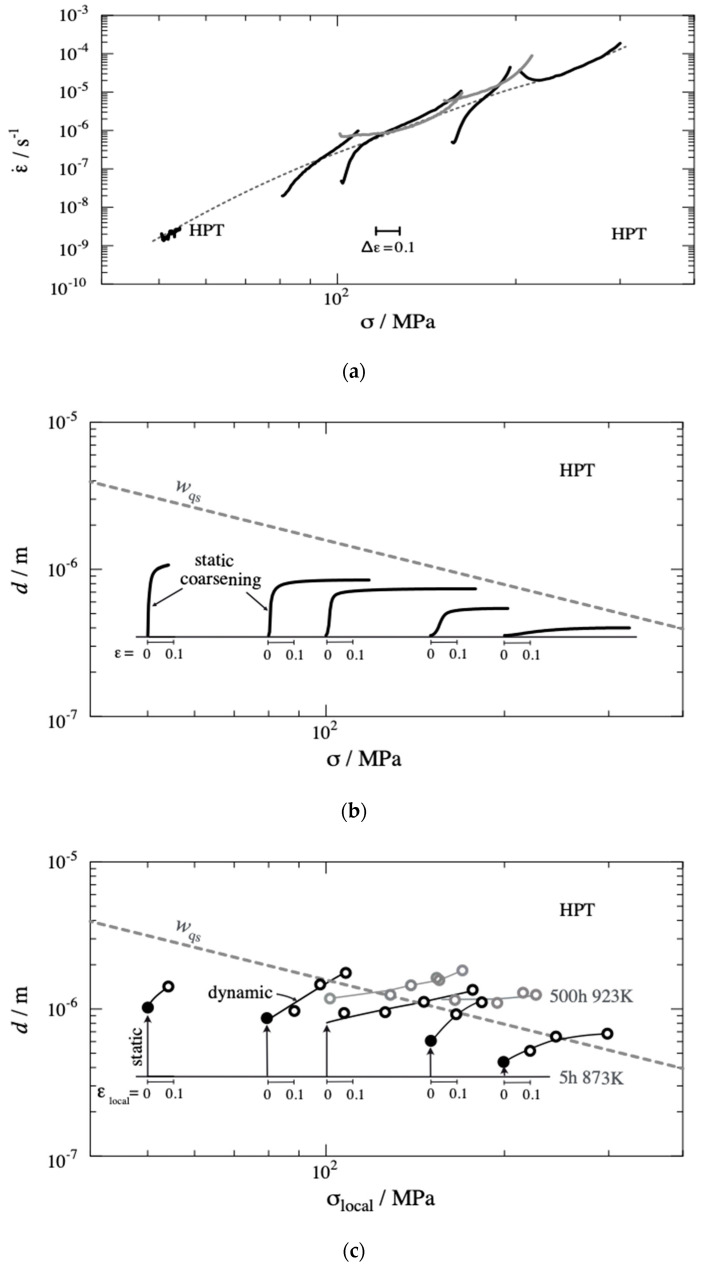
(**a**) Quasi-stationary creep rate vs. stress for CG- and SPD-processed P92 at 873 K and constant load; scale bar marks strain interval of 0.1. (**b**) Static coarsening of grains in tests (**a**) calculated according to Equation (2) (straight line in Figure 14). (**c**) Grain sizes at end of creep tests of (**a**) plotted versus local strain $\varepsilon_{\mathrm{loc}}$ and local stress $\sigma_{\mathrm{loc}}$ in different parts of specimens including grips; dashed line: quasi-stationary spacing value *w*_qs_ = 10 *bG*/σ (shear modulus *G* = 63.5 GPa [25], Burgers vector *b* = 2.48 × 10^−10^ m [26]) of subgrain boundaries shown for comparison. Gray curves in (**a**) and gray symbols in (**c**) refer to specimens that had undergone long-term annealing at 923 K before creep.

According to the foregoing, *d* and *f*_HAGB_ are key parameters to discuss qs deformation strength in creep. Figure 3b has shown that there is a clear grouping: CG and RS initially have *f*_HAGB_ < 0.5 leading to qs subgrain formation within the grains, and HPS15 and HPT initially have *f*_HAGB_ > 0.5 keeping the grains subgrain-free. HPT5 cannot be categorized by mean values only due to its extreme structural heterogeneity. However, the question is not only for the initial value of *f*_HAGB_ but also for its evolution during creep, up to the minimum and beyond. Regarding *w*, the answer is relatively simple as *w* is approaching its *σ*-dependent qs value *w*_qs_ (see Figure 15) within strain intervals in the order of 0.05 to 0.1.

Figure 15a shows the quasi-stationary creep rate as a function of stress and strain, i.e., creep without the primary stage of network hardening. Figure 15b displays static coarsening of grains in different creep tests. It is seen that most of the static coarsening occurs in the early, slow stages of creep. Figure 15c reproduces data for dynamic grain coarsening under stress and strain as observed at the end of the creep tests at different places with different local values of stress ($\sigma_{\mathrm{loc}}$) and strain ($\varepsilon_{\mathrm{loc}}$). There is a clear tendency of increase of d with increasing $\varepsilon_{\mathrm{loc}}$. Additional long-term annealing at 923 K (gray data) did not have significant influence on the dynamic coarsening process. Therefore, it seems that strain is the major parameter driving dynamic grain coarsening (Figure 15).

With the exception of the test at *σ*_0_ = 50 MPa, data for *d* and *w* are available only for fractured specimens. However, it is possible to use non-uniformity of tensile deformation along the gauge and in the transition to the grips to learn about the evolution with strain during creep. If deformation was uniform until final fracture, stress and strain would be constant over the gauge length and obey Equation (1). However, deformation of our long, flat specimens becomes non-uniform so that strain is concentrated near the fracture site while the local strain *ε*_loc_ away from the fracture side is less than the average, *ε*, so that the local stress, *σ*_loc_(*ε*_loc_) (Equation (1)), is lower than the average stress.

It is apparent that grain coarsening is mainly static at low stress σ and creep rates $\dot{\varepsilon }$ and becomes dynamic at high σ and $\dot{\varepsilon }$. In the whole stress range, *f*_HABG_ remains close to 1. That is understandable, as in the severely predeformed HPT *d* does not significantly exceed *w*_qs_ so that there is no drive for LAGB formation within the small grains. One sees, however, from Figure 15 that the tendency for subgrain (LAGB) formation increases with *σ*. At the highest investigated stresses, subgrain structures with LAGB will be formed in large grains. This leads to LAGB strengthening. At even higher *σ*, the difference between CG and HPT will disappear according to the present reasoning. Therefore, the prediction derived from this discussion is that the curves for all variants will merge for high stresses. This is consistent with the observations in Figure 7. With decreasing stress, on the other hand, the fraction of subgrain-free grains will increase and the material behavior will become HAGB controlled.

A question is whether the GB influence discussed above is sufficient to explain creep softening in individual tests leading to the pronounced rate minimum. Such a minimum may be also due to coarsening of particles [27]. This is a diffusion-controlled and, therefore, time-dependent process. It should disappear when creep becomes fast in HPT-processed specimens. Our creep rates do not display that. The contrary appears to be true for HPT in Figure 15a, as the pronounced rate minimum gets even more pronounced when σ increases from 50 to 150 MPa. As such, we consider coarsening of grains an important reason for creep softening causing the pronounced minimum. The model of Blum and Zeng [23] predicts a variation of the quasi-stationary creep in proportion to ${\left(\sigma \sqrt{d}\right)}^{8}$. A rough check indicates that this variation is consistent with the data in Figure 15b. Thus, it seems that grain coarsening can explain the pronounced minimum in HPT.

We now discuss the other material variants. The situation is clear for the creep rate minimum ${\dot{\varepsilon }}_{\min}$ in CG and RS; both have initial *f*_HAGB_ values distinctly less than 0.5. However, in the course of creep, *f*_HAGB_ increases due to subgrain coarsening. Therefore, it may be expected that CG and RS have a pronounced rate minimum and approach the HPT curve. This tendency is observed but the limited ductility, also related with the relatively high stress exponent of qs creep, terminates the creep process and does not allow for a final conclusion. The behavior of HPS5 cannot be explained in detail as not enough is known about the evolution during creep. However, the relatively high initial *f*_HAGB_ value in connection with the relative homogeneity of the structure of HPS5, in contrast to the banded structure of RS (Figure 1), may justify HAGB-controlled behavior of HPS5 similar to HPS15 and HPT.

## 5. Summary and Conclusions

Martensitic creep-resistant P92 steel was processed by different methods of severe plastic deformation (SPD), such as rotation swaging, high-pressure sliding, and high-pressure torsion at room temperature. The creep behavior of SPD-processed P92 steel at 873 K and different stresses were compared with those of coarse-grained P92 steel. The microstructure changes during annealing and creep testing were investigated. The main results are:

1. Severe plastic deformation of P92 at room temperature by different methods led to the increase of imposed strains *ε*_SPD_ from about 1.4 in rotation swaging (RS), 2.6 to 7.9 in high-pressure sliding (HPS), and 25 in high-pressure torsion (HPT), which causes grain refinement. The overall boundary spacing, *w*, including LAGBs and the grain size, *d* (HAGB only), decrease in proportion to $1/\sqrt{\varepsilon_{\mathrm{SPD}}}$ and $1/\varepsilon_{\mathrm{SPD}}$, respectively, until the grains become nearly free from LAGB-based subgrains, and HAGBs dominate.
2. Annealing at 873 K coarsens the grains; the more they coarsen, the smaller the initial grain size. Concurrent creep accelerates coarsening.
3. The minimum creep rate, ${\dot{\varepsilon }}_{\min}$, is much enhanced at stresses where all grains remain subgrain-free. When the stress becomes high enough for subgrains with LAGBs to form, the differences in ${\dot{\varepsilon }}_{\min}$ disappear. Low stress sensitivity of the creep rate in the region of HAGB dominance greatly ductilizes the material.
4. Coarsening of subgrains and grains accompanies softening with creep strain after ${\dot{\varepsilon }}_{\min}$.
5. The heterogeneous microstructure of RS containing large elongated grains with subgrains exhibits the highest creep resistance but the lowest ductility.

The observed creep softening related with grain refinement by SPD indicates that there are only limited means to improve creep resistance. However, the enormous increase in ductility may be a significant advantage in some cases. In any case, the present findings seem to be of relevance for the problem of creep at very low stresses and creep rates in long-term application of tempered martensite Cr steels. Due to inverse stress dependence, the qs subgrain size becomes very large under these conditions so that the grains become more and more subgrain-free and the fraction of LAGBs approaches and exceeds 0.5. In this situation, one must expect that the stress dependence of the creep rate changes compared to the conventional CG case with *d* >> *w* because dynamic recovery is accelerated by HAGB control. This expectation of softening, accompanied by declining stress exponent of creep rate, agrees with many observations made in the range of very slow creep, which so far have not found a satisfactory and generally accepted explanation.

## Figures and Tables

**Table 1:** Predeformation (*ε*_SPD_) imposed by methods of severe plastic deformation (SPD).

Material State of P92 Steel	RS	HPS5	HPS15	HPT
*ε*_SPD_	1.4	2.6	7.9	25 ± 5
